# Garden, greenhouse, or climate chamber? Experimental conditions influence whether genetic differences are phenotypically expressed

**DOI:** 10.1111/plb.70231

**Published:** 2026-05-13

**Authors:** P. Karitter, M. March‐Salas, A. Ensslin, R. Rauschkolb, S. Godefroid, H. Poorter, J. F. Scheepens

**Affiliations:** ^1^ Faculty of Biological Sciences, Plant Evolutionary Ecology Goethe University Frankfurt Max‐von‐Laue‐Str. 13 Frankfurt am Main 60438 Germany; ^2^ Area of Biodiversity and Conservation, Department of Biology and Geology, Physics and Inorganic Chemistry. Universidad Rey Juan Carlos‐ESCET Instituto de Investigación en Cambio Global (IICG) Madrid Spain; ^3^ Conservatory and Botanic Garden of the City of Geneva Geneva Switzerland; ^4^ Institute of Biodiversity, Ecology and Evolution Friedrich Schiller University Jena Jena Germany; ^5^ German Centre for Integrative Biodiversity Research (iDiv) Halle‐Jena‐Leipzig Leipzig Germany; ^6^ Senckenberg Institute for Plant Form and Function Jena (SIP) Jena Germany; ^7^ Meise Botanic Garden Meise Belgium; ^8^ Horticulture and Product Physiology Wageningen University Wageningen the Netherlands; ^9^ Department of Biological Natural Sciences Macquarie University North Ryde New South Wales Australia

**Keywords:** Common garden experiment, genotype × environment interaction (G × E), growth facilities, *Leontodon hispidus*, phenotypic variation, refresher generation, resurrection experiment

## Abstract

- Common‐environment experiments are important to study genetically based phenotypic variation within and among plant populations. Such experiments can be performed in an experimental garden, greenhouse, or climate chamber. However, phenotypic expression may be strongly affected by the environmental conditions and influenced by parental and storage effects. In particular, when the expression of genetically based phenotypic variation depends on the environment (G × E interaction), it can strongly influence conclusions.
- In this study, we assessed the effects of three different growth facilities – outdoor garden, greenhouse, and climate chamber – on phenotypic expression. We compared ancestral and descendant genotypes of the same population of *Leontodon hispidus*. We further evaluated differences in phenotypic expression between plants grown after one (F1) vs. two (F2) intermediate generations.
- As expected, we observed strong differences among plants growing in different environments (*i.e.*, facilities). More importantly, we found that descendants had larger rosettes than ancestors only in the greenhouse and they flowered later than ancestors exclusively in the climate chamber, indicating G × E interactions. We did not find significant differences between different intermediate generations within the growth facilities.
- Overall, our study demonstrates that environmental variation among growth facilities can determine both the presence and magnitude of phenotypic differences. Consequently, differences observed in certain experimental settings may be overestimated compared with expression under natural conditions. Concluding, we recommend combining greenhouse and growth chamber resurrection experiments with field experiments to obtain a more comprehensive understanding of evolutionary changes.

## Graphical abstract

Expression of genetic differences depends on the experimental environment as seen in flowering time differences between ancestors and descendants that emerge in climate chambers but not in greenhouse or garden conditions.

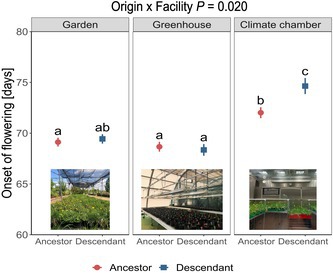

## INTRODUCTION

Phenotypic plasticity is the ability of a given genotype to express different phenotypes depending on the environment (Sultan 2000). Plastic responses to the environment complicate studies on the genetic basis of phenotypic differences, as field observations at population origins cannot be used to unravel genetic from plastic effects on the phenotype. However, by growing plants of the same species from different origins in a common environment, phenotypic variation among plant populations can be attributed to genetic differences (Turesson 1922; Clausen *et al*. 1940; Sultan 2000; Kawecki & Ebert 2004). Common‐environment experiments also lie at the basis of reciprocal transplant studies, which aim to study local adaptation (Clausen *et al*. 1940; Kawecki & Ebert 2004). Furthermore, common‐environment experiments are widely used to study rapid adaptation to recent environmental change. This can be achieved in evolution experiments by applying different selection pressures (*e.g*., water shortage) over multiple generations, followed by a common‐environment test generation (Johnson *et al*. 2022). An alternative method is the resurrection approach, in which seeds collected before a potential selection pressure are revived and compared with plants from a recently collected sample of the same population (Franks *et al*. 2018).

Common‐environment experiments are often performed under artificial conditions that may not reflect natural environments, and plant responses can vary depending on the type of growth facility: outdoor garden, greenhouse or climate chamber (Poorter *et al*. 2016). While differences in trait expression across facilities (*i.e*., main effects of the environment) are expected and generally not concerning, issues arise when the choice of facility influences trait differences among plant origins, potentially leading to contrasting conclusions about origin‐related traits. Such genotype‐by‐environment (G × E) interactions, in which reaction norms of plant origins vary across environments, are well documented (Sultan 2000; Ghalambor *et al*. 2007; Nicotra *et al*. 2010), and reciprocal transplant studies or experiments with intentional environmental treatments that demonstrate plant origin variation in plastic responses can be informative and explain plant local adaptation and plant fitness (Kawecki & Ebert 2004; Matesanz *et al*. 2020). However, when environmental differences among growth facilities are unintended, they may obscure or even reverse trait differences among plant origins.

To address this, methodological studies are needed that investigate whether and how the choice of growth facility influences the detection of plant origin effects—specifically, whether interactions between plant origin and facility (G × E effects) cause these effects to appear or disappear, or even reverse direction (*i.e*., crossover G × E effects). Such studies are essential to assess the reliability of conclusions and to understand the causes of G × E interactions.

Research questions may guide the choice of the experimental environment. Experiments performed in growth facilities offering or mimicking natural conditions may yield different insights than experiments performed under non‐natural conditions or deliberately simulating future or even unrealistic conditions (*e.g*., non‐limiting resources or stress). In particular, growing plants under non‐natural conditions may reveal cryptic genetic variation that remains unexpressed under natural conditions (Ghalambor *et al*. 2007). Thus, depending on the study questions and the experimental setup, studies may either overlook genetic variation that enhances fitness under current natural conditions or, in contrast, detect genetic variation with little current adaptive relevance in the field (although potentially beneficial in the future). Hence, it is important to understand whether and to what extent different growth facilities yield consistent results, or whether they introduce biases in estimates of genetically based phenotypic differences.

Few previous studies have addressed these questions. For instance, Massonnet *et al*. (2010) studied leaf growth variables and other traits of three *Arabidopsis thaliana* genotypes in 10 laboratories and found that modest variations in growing conditions such as temperature, light quality and the handling of the plants can induce significant differences in molecular profiles and phenotypes. Poorter *et al*. (2012) described outdoor experimental gardens as relatively close to natural field conditions, with low spatial but high temporal variability in temperature, light and water supply, frequent plant damage (*e.g*., hail, herbivory, late frost), and occasional extremes such as high irradiance, high temperatures, and drought. In contrast, greenhouses offer buffered environments with heating systems protecting against frost, shading screens to counteract high irradiance, no wind, protection against other external stressors, and controlled water supply (Poorter *et al*. 2012). However, air temperatures can peak depending on the ventilation system, and the plants can be shaded by structural elements of the greenhouse (Cabrera‐Bosquet *et al*. 2016). Climate chambers allow even greater control but introduce artificial conditions that deviate strongly from field conditions. In particular, light can have strong vertical gradients and horizontal heterogeneity in growth chambers (Poorter *et al*. 2012). Therefore, experimental gardens, greenhouses, and growth chambers differ in average conditions and in spatiotemporal variability, affecting plant trait expression.

Besides environmental cues, plant responses can also be confounded by non‐genetic variability induced by parental effects (Latzel *et al*. 2023) or seed storage (Franks *et al*. 2018). Parental effects occur when the parental phenotype or parental environment affects the offspring phenotype (Badyaev & Uller 2009; Auge *et al*. 2017). Seed provisioning is one major parental effect that can impact the offspring, because the resource availability and environmental conditions (*e.g*., light quantity, quality and duration, water availability, temperature, herbivory) of the mother plant can determine the amount of resources invested in the seeds and consequently affect seedling establishment and early life history traits (Herman & Sultan 2011). Other parental effects include hormone‐driven effects on physiology of the seedling or epigenetic processes through passing on distinct DNA methylations or chromatin changes (Herman & Sultan 2011; Richards *et al*. 2017). Although parental effects can have ecological and evolutionary significance (Latzel *et al*. 2023), their influence can be a source of bias in studies on genetic differentiation when parental environmental conditions differ among genotypes. Besides parental effects, storage periods of the seed can affect seed viability and post‐emergence traits (Franks *et al*. 2018). In resurrection experiments for example, where plants from the same population but different generations are compared, storage duration and conditions have likely been different. Consequently, phenotypes of seedlings may express varying plastic responses and they may lead to over‐ or underestimation of the evolutionary change (Weis 2018).

Following best‐practice recommendations for resurrection studies (Franks *et al*. 2018), these biases can be minimized by acclimating experimental plants to common environmental conditions for one or more generations prior to the experiment (*i.e*., a ‘refresher’ or ‘intermediate’ generation). Parental effects and effects of seed storage typically disappear after one generation in a new environment (Agrawal 2002; Gianoli 2002; Franks *et al*. 2018), although persistance across multiple generations has also been reported (Wulff *et al*. 1999). To reduce these effects, Latzel (2015) recommends applying at least two intermediate generations. However, growing intermediate generations is time‐ and labour‐intensive, especially when plants do not flower in their first year, so it is not always implemented in common‐environment studies (Bischoff & Müller‐Schärer 2010; Rauschkolb *et al*. 2023).

In this study, we investigated differences in the phenotypic expression of ancestor and descendant lines (*i.e*., two temporal origins) of a population of the perennial herb *Leontodon hispidus* across common‐environment experiments with potted plants conducted in three growth facilities – an outdoor garden, a greenhouse and a climate chamber – that differ in light, water availability and temperature. We also assessed whether these effects differ between offspring from the first (F1) and second (F2) intermediate generation. To contextualize potential evolutionary changes, we analysed long‐term climate data from a weather station in Maastricht (KNMI, 1991–2022), located approximately 14 km from the *L. hispidus* population. These data showed an increase in mean annual temperature from 10.28 °C in 1995 (ancestor collection) to 11.27 °C in 2018 (descendant collection), alongside a decrease in annual precipitation from 7694.5 mm to 7374.1 mm (Fig. S1). Previous studies on the same population have shown evolutionary shifts toward faster germination, faster growth, decreased specific leaf area (SLA) and increased leaf dry matter content (LDMC) (Rauschkolb *et al*. 2022; Karitter *et al*. 2024, 2025), traits that are likely advantageous under drier conditions, shorter growing seasons and increased competition. Given these environmental changes and prior findings, we expect this population to have evolved trait differences that are detectable across growth facilities, even if absolute trait values vary among environments. Accordingly, we anticipate evolutionary shifts toward drought avoidance, such as faster growth and earlier flowering, as well as drought tolerance, such as decreased SLA and increased LDMC.

Based on our experimental framework using three growth facilities to test for environmental effects and G × E interactions, we hypothesize that (1) average trait values differ among the three growth facilities, (2) genetically based phenotypic differences between ancestors and descendants can be inconsistent across growth facilities (G × E), and (3) one intermediate generation is insufficient to eliminate non‐genetic differences between temporal origins.

## MATERIAL AND METHODS

### Study species

*Leontodon hispidus* (Asteraceae) is a perennial, self‐incompatible, insect‐pollinated herbaceous species and typically flowers from June to October (Kühn & Klotz 2002). It is widespread throughout Europe and commonly found in calcareous grasslands. Seeds were collected from two temporal origins, 1995 (ancestors) and 2018 (descendants), from a single population in a dry calcareous grassland in a Belgian nature reserve (50°47′35″ N, 5°40′25″ E). The distance to the nearest population is approximately 2 km, likely hindering gene flow into the population. The staff of the Meise Botanic Garden (Belgium) collected the ancestral seeds for conservation purposes, and efforts were made to represent the genetic diversity of the population by collecting from as many individuals as possible dispersed throughout the population. The seed material from an unknown number of mother plants was cleaned, bulked, dried at 15% relative humidity, and stored at −20 °C at the seed repository of the Meise Botanic Garden. In the summer of 2018, we revisited the population and estimated the population size at approximately 100 individuals. We collected seeds from 20 mother plants (*i.e*., 20%) of the descendant population. More seeds could not be collected due to time constraints. These seeds were cleaned, bulked, dried and then stored at 4 °C.

### Experimental design

Ancestral and descendant seeds were grown for two consecutive intermediate generations (F1 and F2). The germination rate of the initial seed material (F0) was 30% in ancestors and 80% in descendants (Rauschkolb *et al*. 2022). The low germination rate in the ancestors raises the possibility of ‘invisible fractions’ (Weis 2018), where only a subset of the phenotypes present in the seed bank is expressed. However, Rauschkolb *et al*. (2022) characterized genomic variation of our study population and found similar genomic relatedness and allelic richness among individuals within both temporal origins (ancestors and descendants) without evidence of kinship structure. This supports the comparability of the sampling procedures and indicates that enough mother plants were collected and a representative number of seeds have germinated. For the first intermediate generation (F1), we sowed 300 seeds from each temporal origin and randomly selected 15 individuals from each temporal origin which were randomly pollinated by hand in net cages to prevent unintentional cross‐pollination (Rauschkolb *et al*. 2022). As all available seeds of the F0 generation were used to breed the F1 generation, no seeds remained for our experiment. To grow the F2 generation, we used seeds from the F1 intermediate generation and grew them under similar conditions. As hand pollination of F1 plants had low success in many seed families, we employed bumblebees (*Bombus terrestris*) (Natupol Seeds, Koppert GmbH, Straelen, Germany), which are more effective pollinators. Ultimately, seven maternal lines from the F1 intermediate generation and eight maternal lines from the F2 intermediate generation yielded sufficient seed material for both temporal origins.

In July 2022, we placed 12 pots (1.5 L) per maternal line filled with cultivation soil (Spezial Substrat Typ T1b, Hawita GmbH, Vechta, Germany) in the greenhouse and sowed three seeds into each pot. All pots were watered three times a week to maximum soil capacity. After the seedlings had developed their first true leaf, we thinned them to a single individual per pot, repositioning the remaining seedling to the centre. We measured initial size as rosette diameter and then randomly assigned pots to three groups, each containing four individuals per maternal line. Each group was subsequently grown in a different growth facility in Frankfurt am Main (Germany) for the remainder of the experiment: outdoor garden, greenhouse or climate chamber. In total, we used 360 plants for this experiment (3 growth facilities × 2 temporal origins × 2 generations × 7/8 maternal lines × 4 replicates).

Pots in the garden were placed on gravel 2 m below a shading cloth to reduce radiation and temperature stress (Schattiergewebe 45%, Nitsch GmbH, Kreuztal, Germany). Plants were randomized every 2 weeks and watered weekly to soil capacity for the duration of the experiment. In the greenhouse, the plants were placed 1 m below lamps with a combination of two fluorescent tubes (Lumilux HO 80 W‐865, Osram, Berlin, Germany, and Gro‐Lux FH 80 W, Sylvania, Erlangen, Germany). The lamps were programmed to switch on between 6 am and 8 pm (14 h photoperiod) whenever the natural light intensity went below 360 μmol m^−2^ s^−1^ outside. To avoid extreme temperatures, sliding shutters and lamps were programmed to respond once light intensity surpassed 1100 μmol m^−2^ s^−1^ outside. The climate chamber (ThermoTec GmbH, Weilburg, Germany) was set to a 14 h–10 h day–night cycle with 21 °C during the day and 18 °C during night to simulate the start of the growing season. Air humidity was set to a constant 60% and the plants were placed 1 metre below halogen lamps (Radium HRI‐BT 400 W/D Pro Daylight, Lampenwerk GmbH, Wipperfürth, Germany).

**Fig. 1:**
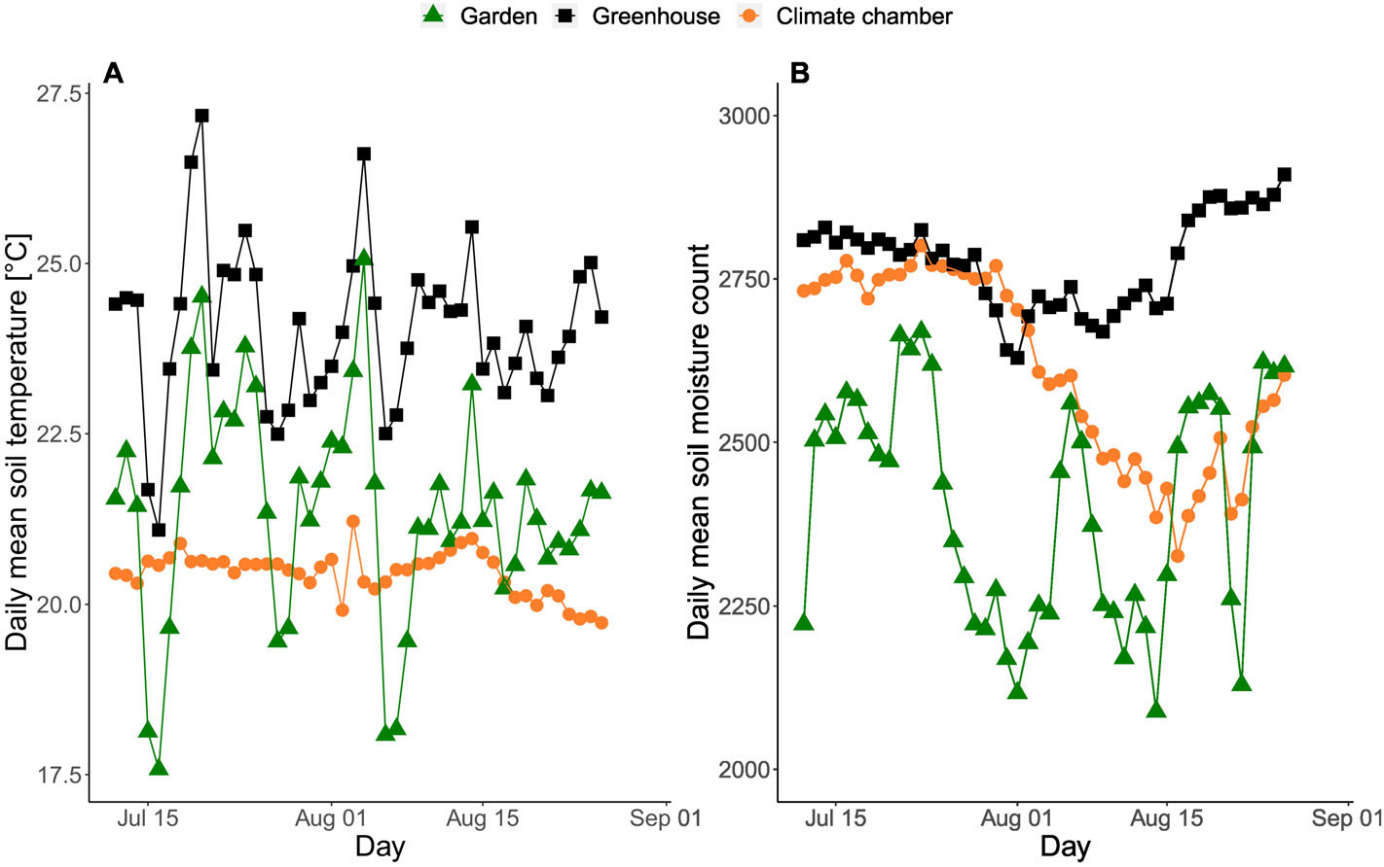
Daily mean soil temperature (A) and daily mean soil moisture count (B) of four random pots grown in different growth facilities. The growth facilities are garden (green triangles), greenhouse (black squares), and climate chamber (orange circles). Soil moisture is measured in *counts*, the unit provided by the TMS‐4 logger.

In each growth facility, we inserted four temperature and soil moisture loggers (TMS‐4 logger, Tomst, Prague, Czech Republic) in the centre of 1.5 L pots with the same cultivation soil and positioned them randomly among the pots with plants. The loggers monitored soil temperature at 5 cm depth, soil surface temperature, air temperature at 5 cm above the soil as well as soil moisture every 15 min. We used these data to calculate mean values of all four loggers from each environment and to derive daily mean values for all parameters (Fig. 1, Fig. S2). Furthermore, we calculated mean values for soil temperature, soil surface temperature, and air temperature over the course of the whole experimental period (Table 1). On average, temperatures were intermediate in the garden with 21.2–22.6 °C, highest in the greenhouse with 23.9–26.1 °C, and lowest in the climate chamber with 20.4–21.2 °C (Table 1).

**Table 1:** Mean values and standard errors of environmental variables over the whole experimental period for all growth facilities.

Growth facility	Temperature [°C]			Daily light integral [mol m^−2^d^−1^]
	Soil (5 cm)	Soil surface	Air	
Garden	21.2 ± 0.2	22.2 ± 0.3	22.6 ± 0.3	20.3 ± 0.8
Greenhouse	23.9 ± 0.2	25.5 ± 0.2	26.1 ± 0.2	3.7 ± 0.1
Climate chamber	20.4 ± 0.05	21.2 ± 0.03	20.7 ± 0.04	15.0 ± 1.0

On the first day of the experiment at noon, we measured the light intensity using a light meter (Panlux electronic 2, GMC‐Instruments, Nürnberg, Germany) in the outdoor garden and greenhouse at 12 spatially distributed pots at the same height 2 cm above the soil. After the end of the experiment, hourly solar radiation data (Wm^−2^) was extracted for the whole experimental period from a weather station in the outdoor garden (iMetos 1, Pessl Instruments GmbH, Weiz, Austria). To assess, how much radiation the plants received in the outdoor garden, we multiplied the solar radiation data by 0.55 to account for the shading cloth (45% shading) and converted the data into photosynthetic photon flux density (PPFD, μmol m^−2^ s^−1^) using the default function *Rg.to.PPFD()* from the *bigleaf* package (Knauer *et al*. 2018) in R (version 4.0.3, R Core Team 2020). The fraction of incoming solar irradiance to photosynthetically active radiation (PAR) was set to 0.5 and the conversion factor was set to 4.6. According to the light intensity measurements at the start of the experiment, the greenhouse received 18.5% of the light that the outdoor garden plants received. We used this ratio to approximate how much PPFD the plants in the greenhouse received over the course of the experiment by multiplying the PPFD data of the outdoor garden by 0.185. We summed all values per day to calculate the daily light integral (DLI) for each day and then calculated the mean DLI for the whole experimental period. For the climate chamber, we directly measured PPFD at 12 spatially distributed spots at pot height using a PAR sensor (PAR Special sensor SKP 210, Skye Instruments Ltd, Powys, UK). Since the photoperiod was constant in the climate chamber (14 h), we multiplied the mean PPFD measurement (297.5 μmol m^−2^ s^−1^) by the photoperiod (in seconds) to calculate the DLI. The average DLI over the course of the experiment (Table 1) was highest in the garden (20.3 mol m^−2^ d^−1^), lowest in the greenhouse (3.7 mol m^−2^ d^−1^) and intermediate in the climate chamber (15.0 mol m^−2^ d^−1^). We did not observe any obvious differences among growth facilities in abiotic or biotic conditions beyond temperature, soil moisture and light availability; in particular, we detected no visible herbivory or fungal damage.

### Measurements of plant functional traits

During the experiment, we recorded onset of flowering three times per week and we regarded a plant as flowering when the first anther was visible. Plants started flowering in August and most plants had flowered after 3 months. At this point, in November, we measured the rosette diameter as a measure of growth and ability to capture light. For each individual, we measured the chlorophyll content of four randomly selected healthy and fully developed leaves in SPAD units using a chlorophyll meter (SPAD‐502 Plus, Konica Minolta, Neu‐Isenburg, Germany). We measured leaf area of three randomly selected healthy and fully developed leaves per plant with the smartphone application ‘Easy Leaf Area’ (Easlon & Bloom 2014). These leaves were dried in a drying oven at 60 °C for 3 days and then weighed together at a fine scale (CPA225D‐0 CE, e = 1 mg, Sartorius AG, Göttingen, Germany). In order to investigate responses in leaf anatomy, we calculated SLA by dividing the combined leaf area of the three selected leaves by their dry weight and calculated the LDMC by dividing the dry weight by the fresh weight. The weight of the three selected leaves was added to the vegetative biomass. Finally, we harvested flower heads and flowering stems as reproductive biomass and leaves as vegetative biomass and dried these using the same procedure. This allows us to determine to what extent plants allocate their resources to vegetative growth versus reproductive structures. Over the course of the experiment, we regularly inspected the plants and found no fungal damage, lice infestations, or significant herbivory damage in any growth facility.

### Data analysis

All statistical analyses were performed using R (version 4.0.3, R Core Team 2020). To analyse rosette diameter, vegetative biomass, SLA, LDMC, onset of flowering, reproductive biomass and the SPAD measurements, we applied linear mixed‐effects models implemented in the *lme4* package (Bates *et al*. 2015) with temporal origin, generation, and environment as fixed factors, as well as their two‐ and three‐way interactions. Maternal line was included as a random factor and initial size as a covariate in all models to reduce potential inaccuracies in the experimental design or procedures (*e.g*., selection of seedlings of different sizes). A two‐way interaction between temporal origin and environment would indicate that ancestors and descendants respond differently from each other depending on the growth facility. A two‐way interaction between temporal origin and generation would indicate that ancestors and descendants differ from each other depending on the intermediate generation. Finally, a two‐way interaction between generation and environment would indicate that the intermediate generations differ from each other depending on the growth facility. When normality and heteroscedasticity of residuals in Gaussian models needed to be improved, we applied the most optimal manually inferred power transformations to these variables (Table 2).

**Table 2:** Results of the statistical models testing the effects of temporal origin (ancestors, descendants), intermediate generation (F1, F2), growth facility (garden, greenhouse, climate chamber), and their interactions on the response variables (y) rosette diameter, vegetative biomass, specific leaf area (SLA), leaf dry matter content (LDMC), onset of flowering, reproductive biomass, and SPAD measurements.

	df	Rosette diameter		Vegetative biomass		SLA		LDMC		Onset of flowering		Reproductive biomass		SPAD	
		F‐value	*P*‐value adj	F‐value	*P‐*value adj	F‐value	*P*‐value adj	F‐value	*P*‐value adj	F‐value	*P*‐value adj	F‐value	*P*‐value adj	F‐value	*P*‐value adj
Initial size		65.42	**<0.001**	25.00	**<0.001**	8.41	**0.020**	17.28	**<0.001**	20.74	**<0.001**	2.09	0.331	0.14	0.792
Origin	1	2.61	0.316	8.55	**0.027**	0.18	0.789	9.39	**0.021**	7.01	**0.046**	0.00	0.969	1.65	0.395
Gen	1	2.26	0.331	0.69	0.617	1.70	0.396	0.23	0.789	1.75	0.396	0.18	0.789	0.25	0.789
Facility	2	155.48	**<0.001**	301.09	**<0.001**	199.31	**<0.001**	91.46	**<0.001**	50.73	**<0.001**	457.85	**<0.001**	280.86	**<0.001**
Origin × Gen	1	0.31	0.789	2.78	0.297	0.23	0.789	1.30	0.457	0.71	0.617	0.20	0.789	1.12	0.521
Origin × Facility	2	3.91	0.067	1.55	0.401	0.52	0.789	0.53	0.789	5.57	**0.020**	2.07	0.320	2.71	0.186
Gen × Facility	2	4.77	**0.034**	0.39	0.789	1.00	0.591	0.68	0.738	0.41	0.789	0.09	0.927	1.98	0.331
Origin × Gen × Facility	2	0.10	0.927	0.32	0.817	0.24	0.851	1.52	0.401	0.30	0.817	1.47	0.412	1.75	0.379
Sample size		348		322		345		345		274		348		350	
Transformation		(x)^1.5^		(x)		log(x)		sqrt(x)		(x)^2^		(x)^0.3^		(x)	

We used linear mixed‐effects models with initial size as covariate and maternal line as random factor followed by ANOVA's. Response variables were transformed if needed to fulfil parametric assumptions. Shown are degrees of freedom (df), F‐values, and adjusted *P*‐values using false discovery rates. Sample sizes are given at the bottom of the table. Significant *P*‐values are shown in bold and marginally significant values are shown in bold.

All models were analysed using the *Anova()* function (package *car*). To test main effects in the presence of interaction terms, we used Type III sums of squares with Satterthwaite‐approximated F‐tests. The resulting *P*‐values (Table S1) were adjusted for multiple testing using the false discovery rate method (Benjamini & Hochberg 1995) via the *fuzzySim* package (Barbosa 2015). Tukey's post‐hoc tests were applied with the *emmeans* package (Lenth 2021) whenever a factor with more than two levels was significant.

## RESULTS

**Fig. 2:**
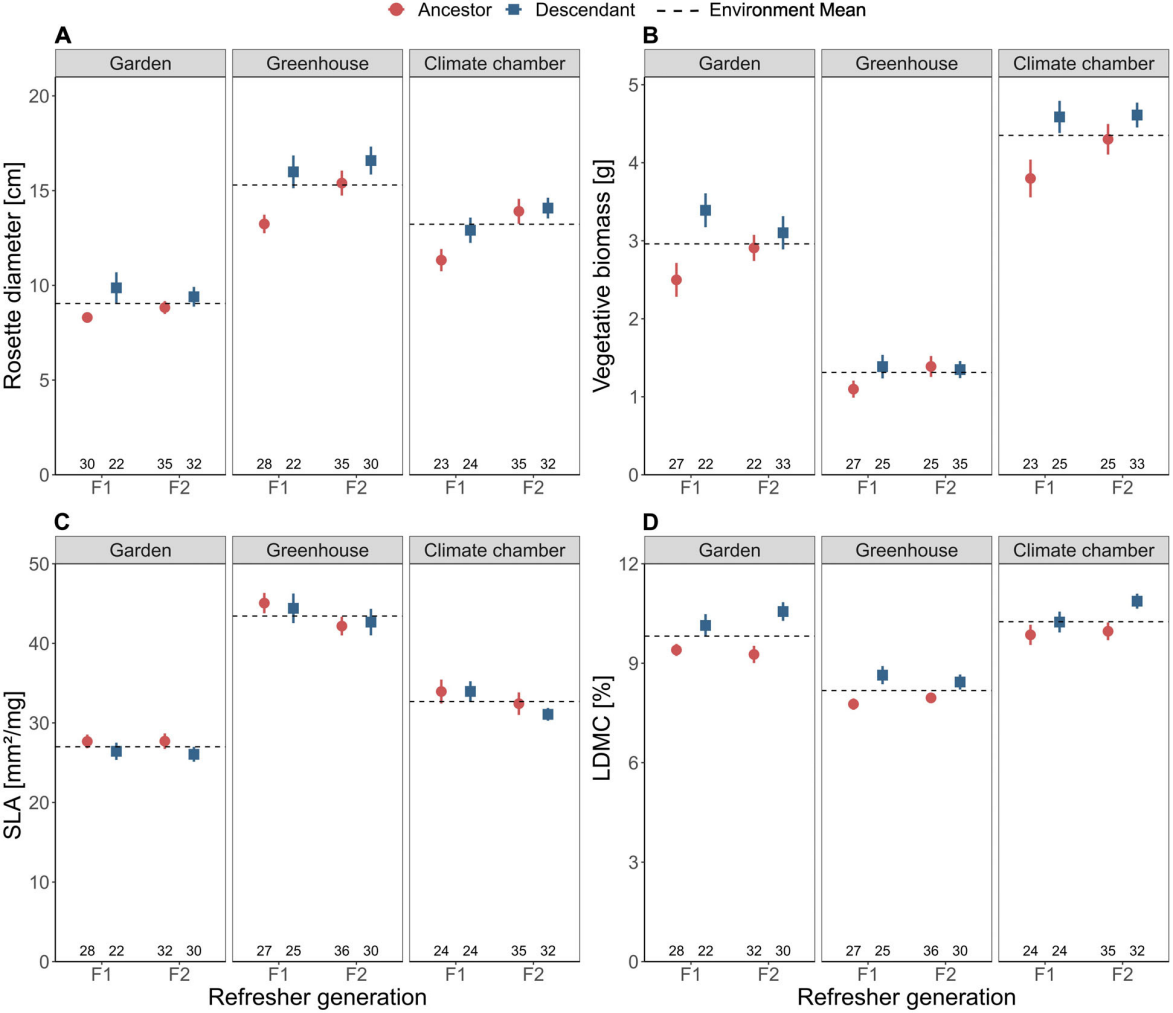
Rosette diameter (A), vegetative biomass (B), specific leaf area (C), and leaf dry matter content (D) of ancestors (blue) and descendants (red) after one intermediate generation (F1) and two intermediate generations (F2) grown in different growth facilities (garden, greenhouse, climate chamber). Shown are means and standard errors. The dotted line represents the overall mean value in each environment. Sample sizes are given at the bottom of the graph below their respective data point.

**Fig. 3:**
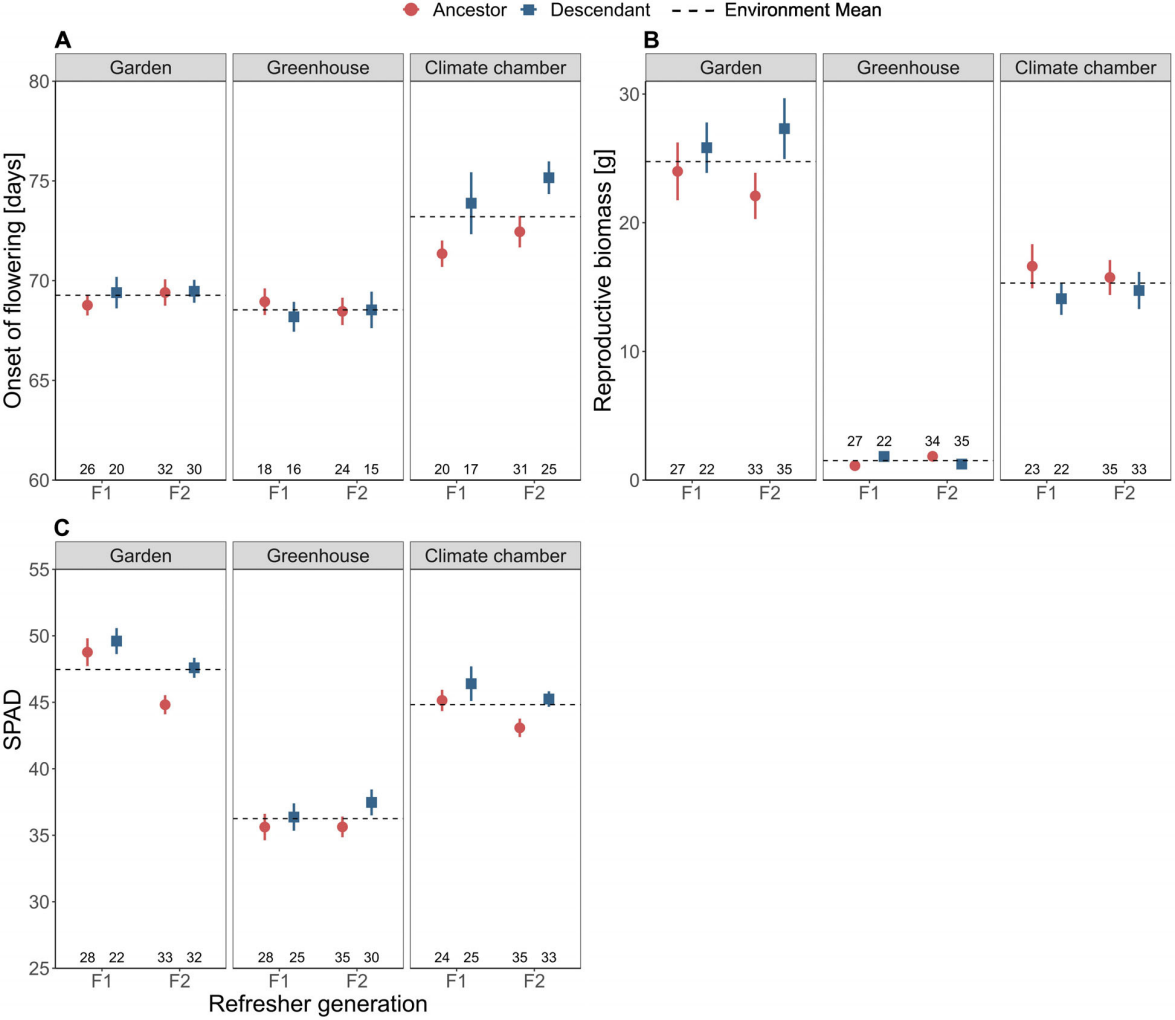
Onset of flowering (A), reproductive biomass (B) and SPAD values (C) of ancestors (blue) and descendants (red) after one intermediate generation (F1) and two intermediate generations (F2) grown in different growth facilities (garden, climate chamber, greenhouse). Shown are means and standard errors. The dotted line represents the overall mean value in each environment. Sample sizes are given at the bottom of the graph below their respective data point. The y‐axes for onset of flowering and SPAD do not start at 0.

The growth facility significantly affected all measured traits (Table 2). In the outside garden, plants had the lowest rosette diameter and SLA, while those traits were highest in the greenhouse and intermediate in the climate chamber (Fig. 2A,C). In contrast, vegetative biomass and LDMC were highest in the climate chamber, followed by the garden and lowest in the greenhouse (Fig. 2B,D). A similar pattern can be observed in the onset of flowering (Fig. 3A): plants in the greenhouse and garden flowered at a similar time, but the onset of flowering of plants in the climate chamber was delayed by approximately 4 days. The reproductive biomass and SPAD values (Fig. 3B,C) were highest in the garden, intermediate in the climate chamber, and markedly low in the greenhouse.

**Fig. 4:**
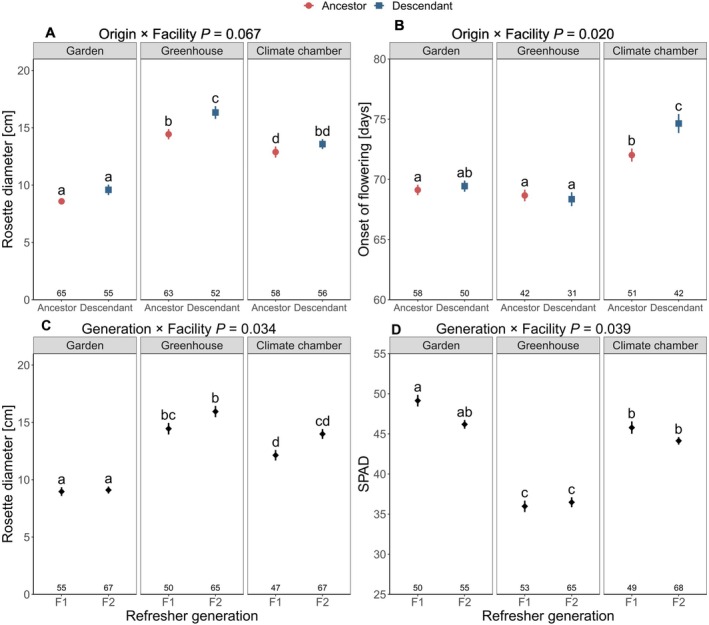
Rosette diameter (A) and onset of flowering (B) of ancestors (red) and descendants (blue) in the different environments (significant Origin × Facility effect). Rosette diameter (C) and SPAD values (D) of F1 and F2 intermediate generations in different facilities (significant Generation × Facility effect). Shown are means and standard errors. Sample sizes are given at the bottom of the graph below their respective data point. The y‐axes for onset of flowering and SPAD do not start at 0. Significant differences (*P* < 0.05) between treatment groups are shown by different letters.

The temporal origin significantly affected vegetative biomass, LDMC, and onset of flowering and we found marginally significant interactions with the growth facility (Origin × Facility) in rosette diameter and significant Origin × Facility interactions in onset of flowering (Table 2). Descendants had on average 13% more vegetative biomass (Fig. S3A) and 8% higher LDMC (Fig. S3B) compared with ancestors, irrespective of the growth facility or intermediate generation. Regarding the rosette diameter, however, descendants had 11% larger rosette diameter (2.1 cm) than ancestors in the greenhouse, while values were similar in the garden and in the climate chamber (Fig. 4A). Descendants also flowered 2.6 days later in the climate chamber than ancestors but flowered at similar times in the garden and greenhouse (Fig. 4B).

The number of intermediate generations did not significantly affect any measured trait consistently across growth facilities, but we found significant interactions using Tukey's post‐hoc tests with the growth facility (Gen × Facility) in rosette diameter and SPAD values (Table 2). The F2 had 11% larger rosettes than the F1 both in the greenhouse and the climate chamber, while the F1 and F2 in the garden were similar (Fig. 4C). Concerning SPAD, the F1 generation showed 3–6% higher values than F2 in the garden and climate chamber, while F1 and F2 did not differ in the greenhouse (Fig. 4D). We found no significant interactions between temporal origin and intermediate generation (Origin × Gen) and no significant three‐way interactions (Origin × Gen × Facility) in the measured traits (Table 2).

## DISCUSSION

We studied the effects of three growth facilities and one *vs* two intermediate generations on the phenotypic expression of ancestor and descendant genotypes of a single population of *Leontodon hispidus*. We found strong phenotypic differences among the three growth facilities. More importantly, we found significant temporal origin × growth facility (G × E) interactions in two traits, indicating that the choice of the growth facility can affect detectability of phenotypic differences. We did not find differences between intermediate generations within the growth facilities, suggesting that there is no need for multiple intermediate generations to sufficiently reduce parental and storage effects for this species.

### Main differences among growth facilities

Plant responses varied among the three growth facilities, and the observed patterns were trait specific. The outdoor garden proved to be the experimental environment where plants were most successful in their reproductive output. Plants in the garden also had comparatively low SLA and high LDMC, which was probably caused in part by the high light availability present at that growth facility (Anten 2005; Poorter *et al*. 2019). Previous studies showed that leaf traits can strongly respond to water availability, with increasing dryness leading to decreasing SLA and increasing LDMC (Poorter *et al*. 2009; Vitra *et al*. 2019). Accordingly, pots in the garden had the lowest soil moisture content (Fig. 1B) and high variability in soil moisture, which was caused by the exposure to natural rain events, high temperature fluctuations (Fig. 1A), and potentially higher evaporation due to wind exposure.

In the greenhouse, plants developed a large rosette diameter and exhibited high SLA. This response is in line with a strategy to increase surface area to improve light capture in low‐light environments (Poorter *et al*. 2019). Indeed, the greenhouse had the lowest light availability (Table 2), but also very high temperatures (Fig. 1A). These high temperatures are also likely to contribute to the high SLA, as they facilitate cell expansion (Atkin *et al*. 1996; Poorter *et al*. 2009). The reproductive biomass and SPAD values were very low, indicating that the plants in the greenhouse had the lowest fitness. Heat stress has been found to have negative effects on sexual reproduction in plants, possibly explaining the lower reproductive biomass in the greenhouse (Tushabe *et al*. 2023). Plants growing in the climate chamber had intermediate rosette diameter, SLA, SPAD values, and reproductive traits, which correlates well with the intermediate light availability (Table 1). Overall, it is not surprising that plants in the different growth facilities responded so strongly, as the differences in abiotic parameters were large (*i.e*., temperature difference = 6 °C, fivefold difference in irradiation) and larger than those reported in comparable studies (Massonnet *et al*. 2010).

### Consistency of trait variation between ancestors and descendants among growth facilities

Although we found that different growth facilities cause plants to differ in their overall performance, it may also be assumed that origin effects would show qualitatively similar results across environments. Under this assumption, if a common‐environment experiment would be performed in a single environment, the expected patterns should be observed irrespective of the common environmental conditions. Three alternative scenarios are, however, possible. First, origin effects may only emerge under specific environmental conditions and not in others, because novel or stressful environments can reveal cryptic genetic variation or amplify differences among plant origins (Hoffmann & Merilä 1999; Ghalambor *et al*. 2007; Schlichting 2008; Paaby & Rockman 2014). This would imply that certain experimental setups may fail to reveal origin effects. Second, origin effects could consistently point in the same direction across environments but vary in strength due to environment‐dependent amplification or compression of genetic variation (Hoffmann & Merilä 1999). Third, origin effects may differ in direction depending on the experimental conditions, that is, the reaction norms of the plant origins cross (Clausen *et al*. 1940; Anstett *et al*. 2021). Consequently, in all three scenarios, the choice of experimental environment could lead to contrasting conclusions.

In our study, descendants consistently had higher vegetative biomass and higher LDMC compared with ancestors irrespective of the growth facility. These results are in line with the previous finding that this population of *L. hispidus* evolved faster growth in recent decades (Karitter *et al*. 2025), which was observed in an experiment in the same greenhouse but conducted in autumn rather than in summer. Furthermore, high LDMC has been associated with increased drought survival (Bongers *et al*. 2017; De La Riva *et al*. 2017), a trait likely to be favoured under the decreasing precipitation and increasing temperatures observed at the population site in recent decades (Fig. 1). LDMC correlates well with strong cell walls and may be beneficial to maintain turgor under drought conditions (Monson & Smith 1982). Therefore, high LDMC could have evolved in this population through selection under increasing drought events linked to climate change (IPCC 2018), although fitness‐based evidence would be required to verify this mechanism.

While the phenotypic differences between ancestors and descendants in vegetative biomass and LDMC were consistent throughout the experimental environments, we found interactions of temporal origin with the experimental environment (G × E) for rosette diameter and onset of flowering, expressed as presence–absence effects. Descendants had a larger rosette diameter in the greenhouse compared with ancestors, but temporal origins did not differ in the other two environments. Given that the most prominent distinction of the greenhouse was its relatively low‐light irradiance, this may have triggered an increase in rosette diameter to capture more light. The fact that the descendants reacted more plastically to low‐light availability can be explained by possible evolutionary processes in recent decades. At the collection site, we observed that grass competitors of *L. hispidus* can substantially shade the rosettes. Combined with high nutrient depositions from the atmosphere (Newman 1995; Galloway *et al*. 2008; Bobbink *et al*. 2010) and surrounding agriculture, *L. hispidus* might have faced strong selection pressure through high competition and may have adapted its ability to plastically respond to increasingly shaded conditions (Karitter *et al*. 2024). However, this explanation needs further testing by additional experiments that include shading treatments. Importantly, this relevant discovery may have been missed if the experiment would have been conducted in the climate chamber or outdoor garden.

Descendants generally flowered later than ancestors exclusively in the climate chamber, where flowering was delayed overall compared with the other environments; however, descendants showed a significantly stronger delay than ancestors. In a previous resurrection study on the same population conducted in a greenhouse, descendants also flowered later than ancestors (Rauschkolb *et al*. 2022), which was explained by the introduction of sheep grazing in 2007, that is, between the sampling of ancestors and descendants, which likely selected for later‐flowering plants. We could neither confirm this result in the greenhouse nor in the outside garden, suggesting that the experimental environments differed in the cues regulating flowering onset. The onset of flowering in herbaceous species depends strongly on environmental cues such as temperature (Capovilla *et al*. 2015) and photoperiod (Rauschkolb *et al*. 2023), which varied among growth facilities. Consequently, the comparability of studies investigating flowering onset across different growth conditions and origins can be limited.

One limitation to our study is the relatively low number of mother plants sampled in the field and used in the study. This may generally impact the comparison between ancestors and descendants and also influence the responses to the different growth environments depending on how well the sampled genetic variation reflects the genetic variation in the population of origin.

### Intermediate generations

We found no differences between the two intermediate generations used in this study across the three growth facilities, and the two intermediate generations also did not differ in their expression of phenotypic variation between ancestors and descendants. These results indicate that one intermediate generation is sufficient to effectively reduce parental and storage effects. Furthermore, the findings confirm the general guidelines for conducting resurrection studies (Franks *et al*. 2018) and show that the labour‐intensive and time‐consuming cultivation of multiple refresher generations is not necessary. Interestingly, the interaction between generation and growth facility (analogous to G × E) for rosette diameter and SPAD reveals that differences between the F1 and F2 generations appear in some facilities but disappear in others. The observed patterns reflect a presence–absence or scale effect rather than a crossover interaction, indicating that generational responses vary in magnitude but do not reverse direction across environments.

However, our results do not inform us whether significant parental effects were present in the F0 generation. The magnitude and nature of parental effects can be strongly dependent on the environmental stresses experienced by the parents (Latzel *et al*. 2023) and are context‐ and species‐specific. It is likely that environmental stresses differed between ancestors and descendants of our study population, as climate change increased the frequency and duration of droughts and heatwaves (IPCC 2018). Any parental effects present were likely eliminated in the first intermediate generation, as shown in other studies (Agrawal 2002; Gianoli 2002). However, because seed material of the originally collected F0 generation was unavailable, we cannot quantify how much the first intermediate generation reduced parental and storage effects.

### Choice of growth facility

The majority of resurrection studies investigate the evolutionary responses of plant populations only in a single growth facility. As our study shows, depending on the trait, phenotypic differences are not guaranteed to be detected in a given experimental environment, even if genetic variation for these phenotypic differences is present. Studying phenotypes under natural field conditions allows us to observe traits as they would occur in the wild. In contrast, providing non‐limiting resources or applying stress can reveal genetic differences that are only expressed under unrealistic or stressful conditions, respectively. This approach may help uncover cryptic genetic variation that could be expressed under future conditions, such as those arising from climate change. However, caution is needed, as overly extreme treatments may elicit responses that would not be expressed under naturally occurring extremes (Ghalambor *et al*. 2007).

The optimal choice among the three growth facilities tested in this study depends on the research objectives. Gardens best mimic natural conditions, while greenhouses and climate chambers, with more divergent conditions, proved more effective in detecting evolutionary changes between ancestors and descendants, as well as between F1 and F2 generations. Low temporal variation in experimental conditions provided in greenhouses and, especially, climate chambers is important to provide similar conditions throughout all ontogenetic stages, and to avoid interactions of ontology and the environmental conditions. However, these two facilities provide limited space which can greatly affect light competition among plants as well as the sample size and thus enforce a trade‐off between control over the environment and statistical power. Resurrection studies are commonly performed in greenhouses, where environmental variation can be minimized to enhance the detection of genetic change (*e.g*., Nevo *et al*. 2012; Hamann *et al*. 2018; Lambrecht *et al*. 2020; Anstett *et al*. 2021; Gay *et al*. 2022). We propose that resurrection experiments conducted under more natural conditions should increasingly be used to confirm patterns identified in controlled experiments and assess local adaptation underlying observed phenotypic changes (Karitter *et al*. 2024; White *et al*. 2025). Phenotypic variation expressed primarily under non‐natural experimental conditions may reflect standing genetic variation shaped by mutation, genetic drift, and context‐dependent selection, and could become relevant under future climates (Ghalambor *et al*. 2007; Schlichting 2008; Paaby & Rockman 2014). In contrast, phenotypic variation expressed under natural conditions is more likely to reflect past or ongoing selection, although it may still be constrained by genetic architecture, trade‐offs and G × E interactions (Kawecki & Ebert 2004; Ghalambor *et al*. 2007).

## CONCLUSION

Our study showed that the choice of growth facility in common‐environment experiments can influence the expression of phenotypic differences among genotypes and thereby affect the conclusions drawn. Thus, studying the evolution of plant populations in only a single environment may lead to incomplete or biased interpretations for some traits. Hence, it is important to carefully select the growth facility or to use multiple facilities, depending on the study aims. In addition, growing a second intermediate generation, rather than only one, did not impact the genetic differences between ancestors and descendants across growth facilities, suggesting that a single intermediate generation is sufficient to reduce detectable parental and storage effects, if present. However, slight differences among growth facilities in the expression of intermediate‐generation effects indicate that parental effects may be overlooked under specific experimental conditions.

Our study examined only a single species from one source population, and the observed patterns may differ among species and populations. Thus, experiments including multiple species and populations are needed to advance our understanding of how growth facilities and the number of intermediate generations influence common‐environment studies of evolution‐driven phenotypic differentiation. Overall, our results support the view that greenhouse and growth chamber resurrection experiments should be complemented by field experiments to achieve a more comprehensive understanding of evolutionary change.

## AUTHOR CONTRIBUTIONS

PK, MMS, and JFS designed the study. SG and RR provided the seed material. PK conducted the experiment. PK, MMS, and JFS analysed the data. PK wrote the first draft and all the authors contributed to further versions of the manuscript.

## CONFLICT OF INTEREST STATEMENT

The authors have no conflict of interest to declare.

## Supporting information


**Fig. S1.** Climate data from a weather station in Maastricht, located 14 km from the *L. hispidus* population, showing (A) average yearly temperature [°C] and (B) yearly precipitation [mm]. The solid black lines represent linear trends (regression lines) over the entire data range. The dashed vertical lines indicate the years in which seeds were collected: 1995 for ancestors and 2018 for descendants.
**Fig. S2**. Daily mean soil surface temperature (A) and daily mean air temperature (B) of four random pots grown in different growth facilities. The growth facilities are garden (green triangles), greenhouse (black squares), and climate chamber (orange circles).
**Fig. S3**. Vegetative biomass (A) and LDMC (B) of ancestors and descendants (significant Origin effect). Shown are means and standard errors. Standard errors of LDMC are too small to be visible. Sample sizes are given at the bottom of the graph below their respective data point.
**Table S1**. Results before adjustments of the statistical models testing the effects of temporal origin (ancestors, descendants), intermediate generation (F1, F2), growth facility (garden, greenhouse, climate chamber), and their interactions on the response variables (y) rosette diameter, vegetative biomass, specific leaf area (SLA), leaf dry matter content (LDMC), onset of flowering, reproductive biomass, number of stems and SPAD measurements. We used linear mixed‐effects models with initial size as covariate and maternal line as random factor followed by ANOVA's. Significant values (*P* < 0.05) are shown in bold.

## Data Availability

The data that support the findings of this study are available from Dryad [https://doi.org/10.5061/dryad.qz612jmx5].
