# Supplementary material for: Garden, greenhouse, or climate chamber? Experimental conditions influence whether genetic differences are phenotypically expressed
Source: Plant Biol (Stuttg). 2026 May 13;28(5):1361–72. doi: 10.1111/plb.70231 (PMC13358643; doi:10.1111/plb.70231)
Supplement: Supplementary file 1 — Fig. S1. Climate data from a weather station in Maastricht, located 14 km from the L. hispidus population, showing (A) average yearly temperature [°C] and (B) yearly precipitation [mm]. The solid black lines represent linear trends (regression lines) over the entire data range. The dashed vertical lines indicate the years in which seeds were collected: 1995 for ancestors and 2018 for descendants. Fig. S2. Daily mean soil surface temperature (A) and daily mean air temperature (B) of four random pots grown in different growth facilities. The growth facilities are garden (green triangles), greenhouse (black squares), and climate chamber (orange circles). Fig. S3. Vegetative biomass (A) and LDMC (B) of ancestors and descendants (significant Origin effect). Shown are means and standard errors. Standard errors of LDMC are too small to be visible. Sample sizes are given at the bottom of the graph below their respective data point. Table S1. Results before adjustments of the statistical models testing the effects of temporal origin (ancestors, descendants), intermediate generation (F1, F2), growth facility (garden, greenhouse, climate chamber), and their interactions on the response variables (y) rosette diameter, vegetative biomass, specific leaf area (SLA), leaf dry matter content (LDMC), onset of flowering, reproductive biomass, number of stems and SPAD measurements. We used linear mixed‐effects models with initial size as covariate and maternal line as random factor followed by ANOVA's. Significant values (P < 0.05) are shown in bold. [file PLB-28-1361-s001.docx]

**Supplement material**


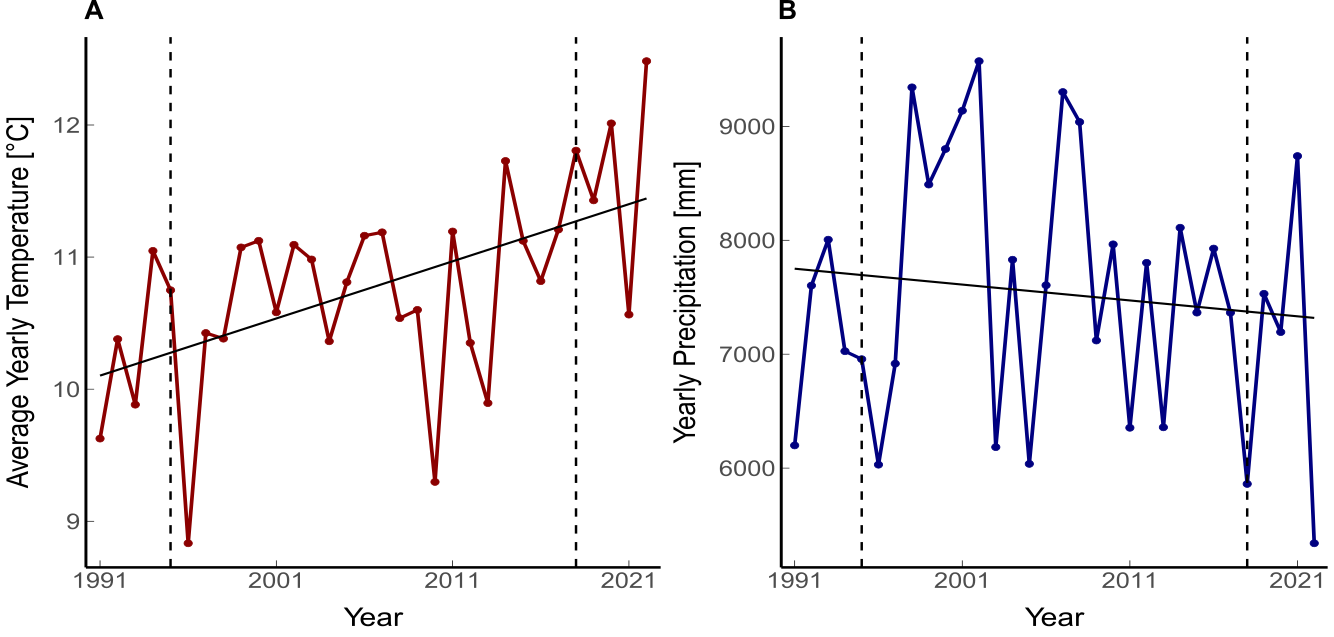


**Figure S1.** Climate data from a weather station in Maastricht, located 14 km from the *L. hispidus* population, showing (A) average yearly temperature [°C] and (B) yearly precipitation [mm]. The solid black lines represent linear trends (regression lines) over the entire data range. The dashed vertical lines indicate the years in which seeds were collected: 1995 for ancestors and 2018 for descendants.


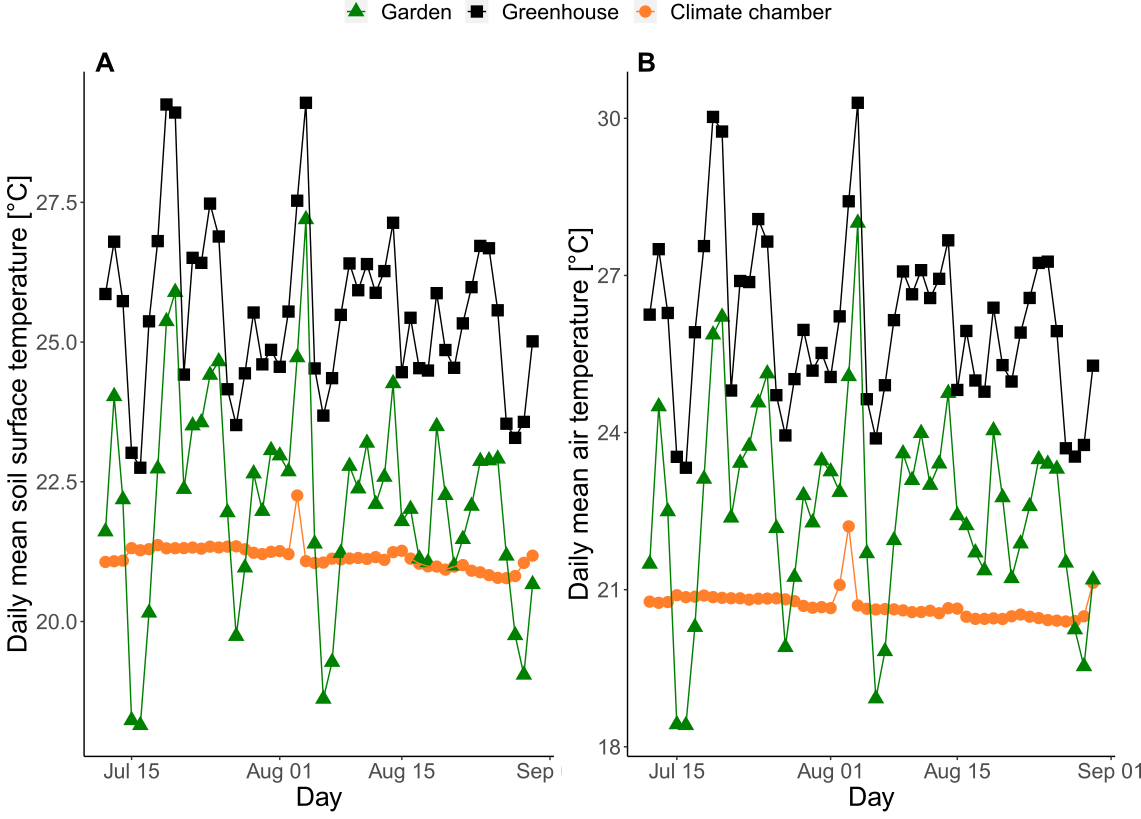


**Figure S2.** Daily mean soil surface temperature (A) and daily mean air temperature (B) of four random pots grown in different growth facilities. The growth facilities are garden (green triangles), greenhouse (black squares), and climate chamber (orange circles).

**
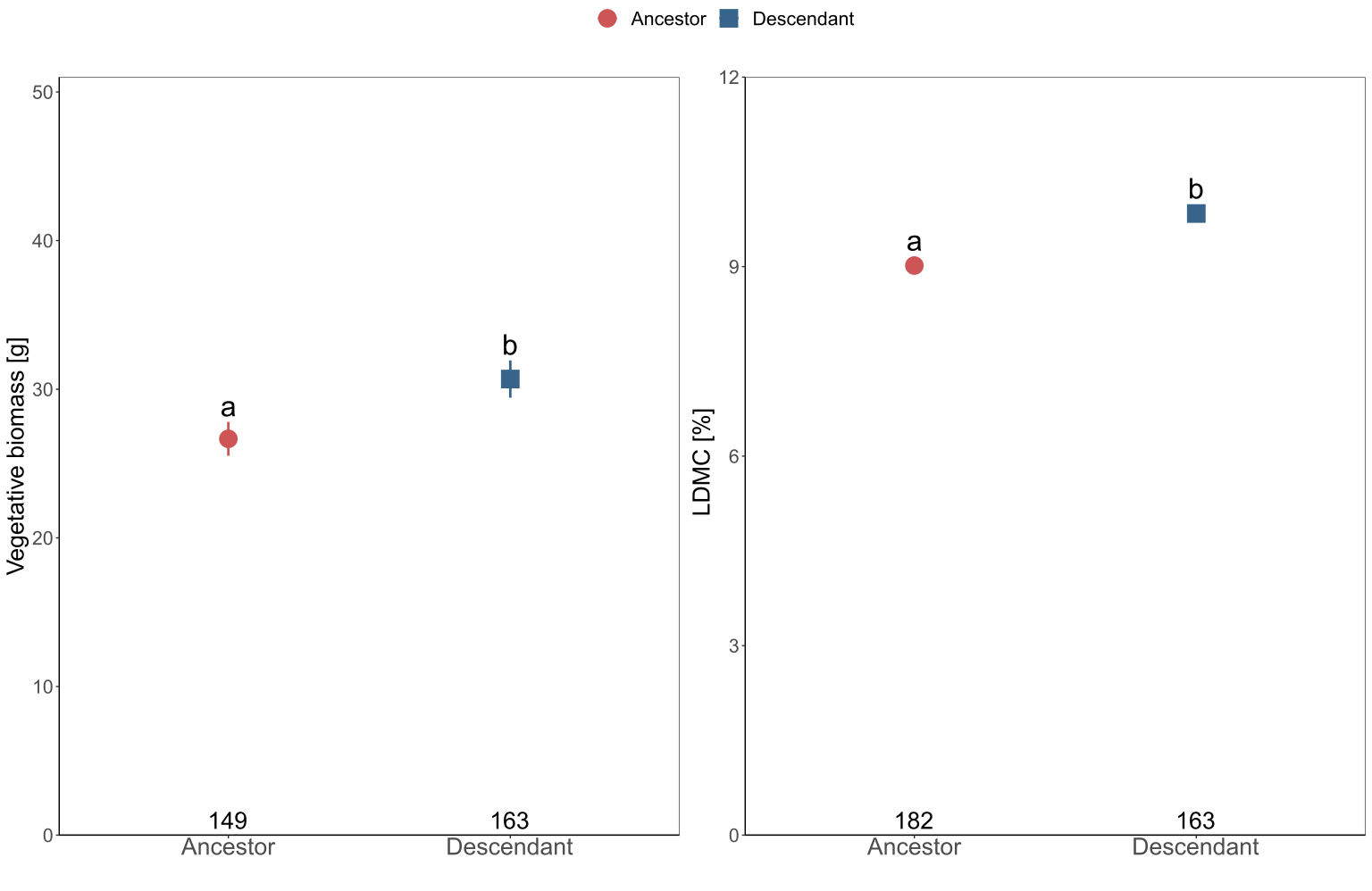
Figure S3.** Vegetative biomass (A) and LDMC (B) of ancestors and descendants (significant Origin effect). Shown are means and standard errors. Standard errors of LDMC are too small to be visible. Sample sizes are given at the bottom of the graph below their respective data point.

**Table S1.** Results before adjustments of the statistical models testing the effects of temporal origin (ancestors, descendants), intermediate generation (F1, F2), growth facility (garden, greenhouse, climate chamber) and their interactions on the response variables (y) rosette diameter, vegetative biomass, specific leaf area (SLA), leaf dry matter content (LDMC), onset of flowering, reproductive biomass, number of stems and SPAD measurements. We used linear mixed effects models with initial size as covariate and maternal line as random factor followed by ANOVA’s. Significant values (p < 0.05) are shown in bold.

|  |  | **Rosette diameter** | **Vegetative biomass** | **SLA** | **LDMC** | **Onset of flowering** | **Reproductive biomass** | **SPAD** |
| --- | --- | --- | --- | --- | --- | --- | --- | --- |
|  | *df* | *p* value | *p* value | *p* value | *p* value | *p* value | *p* value | *p* value |
| **Initial size** |  | **<0.001** | **<0.001** | **0.004** | **<0.001** | **<0.001** | 0.15 | 0.379 |
| **Origin** | 1 | 0.118 | **0.007** | 0.676 | **0.005** | **0.014** | 0.969 | 0.101 |
| **Gen** | 1 | 0.145 | 0.414 | 0.204 | 0.639 | 0.198 | 0.673 | 0.130 |
| **Env** | 2 | **<0.001** | **<0.001** | **<0.001** | **<0.001** | **<0.001** | **<0.001** | **<0.001** |
| **Origin × Gen** | 1 | 0.584 | 0.106 | 0.633 | 0.264 | 0.408 | 0.659 | 0.339 |
| **Origin × Env** | 2 | **0.021** | 0.214 | 0.593 | 0.590 | **0.004** | 0.128 | 0.899 |
| **Gen × Env** | 2 | **0.009** | 0.678 | 0.371 | 0.507 | 0.661 | 0.912 | **0.011** |
| **Origin × Gen × Env** | 2 | 0.902 | 0.728 | 0.785 | 0.219 | 0.740 | 0.232 | 0.817 |
